# Bullous pemphigoid in patients receiving peritoneal dialysis: a case series and a literature survey

**DOI:** 10.1080/0886022X.2021.1910045

**Published:** 2021-04-05

**Authors:** Kohkichi Morimoto, Tadashi Yoshida, Naoki Washida, Kiyotaka Uchiyama, Takashin Nakayama, Hiroshi Itoh, Mototsugu Oya

**Affiliations:** a Apheresis and Dialysis Center, School of Medicine, Keio University, Tokyo, Japan; b Department of Nephrology, School of Medicine, International University of Health and Welfare, Otawara, Japan; c Division of Endocrinology, Metabolism and Nephrology, Department of Internal Medicine, School of Medicine, Keio University, Tokyo, Japan; d Department of Urology, School of Medicine, Keio University, Tokyo, Japan

**Keywords:** Bullous pemphigoid, peritoneal dialysis, hemodialysis, incidence

## Abstract

Bullous pemphigoid (BP) is an autoimmune subepidermal blistering disease. Although several cases of BP in end-stage renal disease patients receiving peritoneal dialysis (PD) or hemodialysis have been reported, the incidence of BP in these patients remains unknown. We recently experienced three PD patients diagnosed with BP. The skin injury was likely to be a trigger of BP in all the three PD patients. Nifedipine and icodextrin exposures were possible factors directly or indirectly affecting the onset of BP, because they were common in the three cases. We also report that the incidence of BP in PD patients was 3/478.3 person-years in a single-center 10-year study. This case series with a literature survey describes that the skin and tissue injuries are potential triggers responsible for the onset of BP in dialysis patients and that the incidence of BP in these patients seems to be much higher than that in the general population.

## Introduction

Bullous pemphigoid (BP) is an autoimmune subepidermal blistering disease [1–3]. The disease presents large (1–3 cm), tense, serous, or hemorrhagic blisters appearing on erythematous, urticarial, or eczematous lesions or apparently normal skin in the extremities and the trunk [1–3]. The bullous lesions can also start with only urticarial-like lesions in some cases [3]. The blisters evolve into eroded and crusted lesions and then heal with postinflammatory changes. Thus far, 12 cases of BP have been reported in patients receiving hemodialysis (HD) [4–14], and only two in patients receiving peritoneal dialysis (PD) [15,16]. Recently, we experienced three cases of BP in our PD patients. Here, we present these three cases and summarize the clinical features of dialysis patients complicated with BP to discover possible factors affecting the disease development. We also calculate the incidence of BP in PD patients in our single institution.

## Case presentation

### Case 1

**Figure 1.**
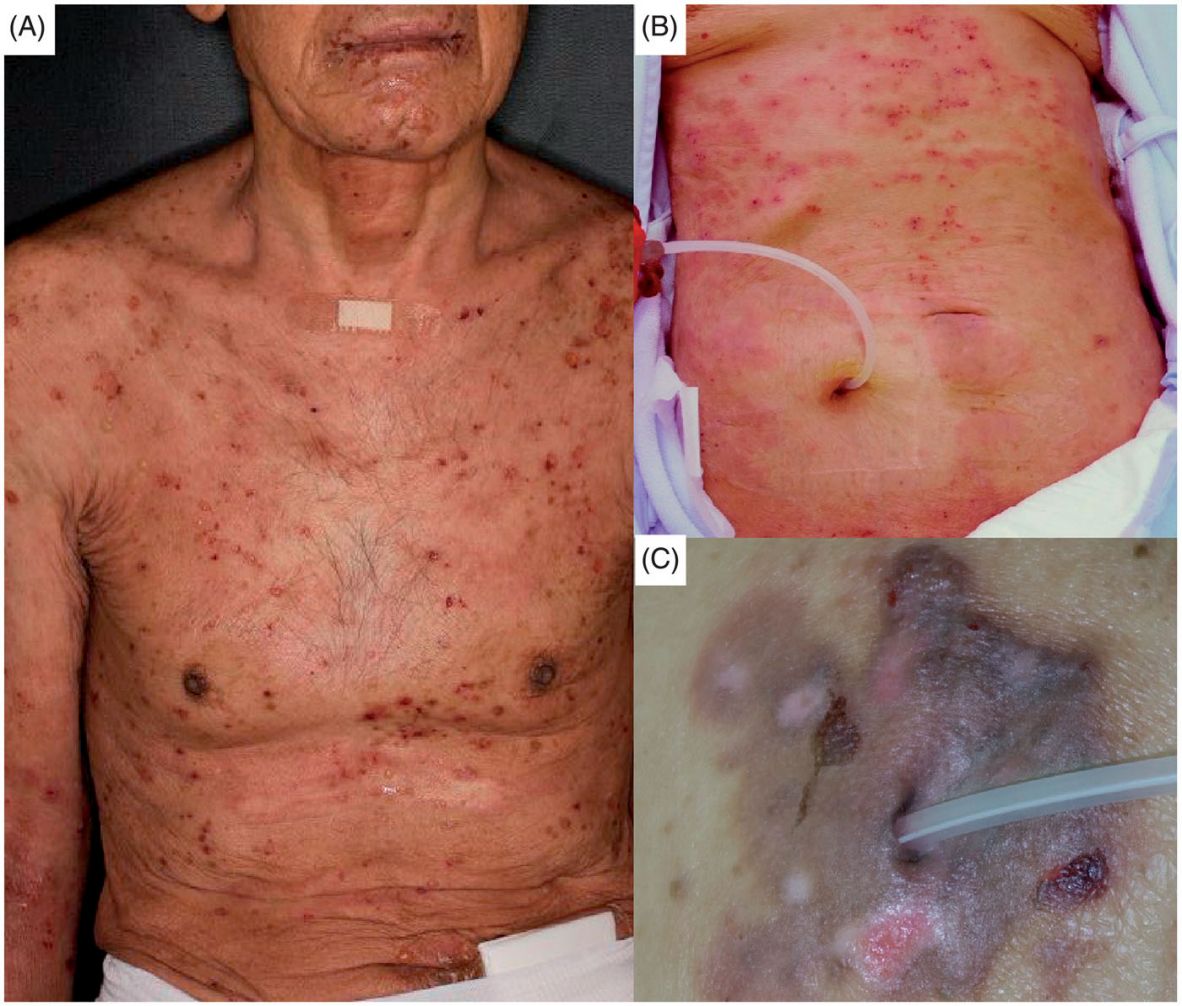
Skin lesions of Case 1 (A), Case 2 (B), and Case 3 (C).

A 72-year-old man with ESRD of unknown cause initiated PD in 2013. He could not choose HD, because he had Buerger disease and preparation of the vascular access on his upper limb was quite difficult. PD catheter was placed using a conventional method in February, and PD was initiated in April using 1.36% glucose solution for a daytime exchange and icodextrin solution for a night exchange. Four weeks after the induction of PD, he presented with pruritic eruptions on his hands. An icodextrin-induced allergic reaction was suspected. He stopped using it, but the pruritic skin lesion deteriorated to diffuse systemic blisters and erosions on his trunk and extremities in a week (Figure 1(A)). The laboratory data, PD condition, and PD prescriptions at the onset are shown in Table 1. The autoantibody for BP was positive (anti-BP180-NC16a antibody 143.9 U/mL [normal range <8.9 U/mL]), whereas the autoantibodies for pemphigus were negative (anti-Dsg1 antibody <3.0 U/mL and anti-Dsg3 antibody <3.0 U/mL). The results of skin biopsy showed subepidermal blister formation and eosinophil infiltration accompanied by the deposition of BP-specific autoantibody; the findings were compatible with BP. He was admitted to our hospital and treated with oral prednisolone (PSL) 1 mg/kg (60 mg) daily for 2 weeks to improve his skin lesions. Then, PSL was tapered (−10 mg/2 weeks) without recurrence. Three weeks after the initiation of PSL administration, an elevation in liver enzymes (aspartate transaminase 149 IU/L and alanine transaminase 145 IU/L), a low platelet count (5.0*10^4^/µL), and atypical lymphocytes in drained peritoneal dialysate were observed. PCR analysis detected high levels of viral DNA (herpes simplex virus [HSV] 6 × 10^7^ copies/mL and varicella-zoster virus [VZV] 2 × 10^2^ copies/mL) in his blood. He was diagnosed with disseminated HSV and VZV infections. Acyclovir at a dosage of 3.5 mg/kg daily was administered, but his general condition deteriorated. Unfortunately, he also developed invasive pulmonary aspergillosis and septic disseminated intravascular coagulation caused by *Enterococcus faecium*. He died in July 2013.

**Table 1.** Laboratory data, PD condition, and PD prescriptions at the onset of BP.

	Case 1	Case 2	Case 3
Urea nitrogen (mg/dL)	70.2	57.8	61.5
Creatinine (mg/dL)	12.1	8.4	18.7
Potassium (mEq/L)	5.2	4.4	4.9
Calcium (mg/dL)	8.1	8.9	9.1
Inorganic phosphorus (mg/dL)	6.6	4.7	7.3
Total protein (g/dL)	6.2	6.3	6.1
Albumin (g/dL)	3.1	2.9	3.4
Red blood cells (/µL)	355 × 10^4^	436 × 10^4^	367 × 10^4^
Hemoglobin (g/dL)	9.9	11.1	11.4
White blood cells (/µL)	7900	8400	5600
Eosinophils (%)	11.0	8.2	7.1
Platelets (/µL)	16.1 × 10^4^	27.1 × 10^4^	15.5 × 10^4^
Intact-parathyroid hormone (pg/mL)	314	170	207
β2-Microglobulin (µg/L)	27.66	27.47	30.45
Anti-BP180-NC16a antibody (U/mL)	143.9	45.8	113.0
Anti-Dsg1 antibody (U/mL)	<3.0	<3.0	<3.0
Anti-Dsg3 antibody (U/mL)	<3.0	<3.0	<3.0
wrKt/V / wpKt/V / wtKt/V	0.42/0.77/1.19	0.43/1.35/1.78	0.16/1.49/1.65
rCcr (mL/min)	3.6	2.4	3.2
pCcr (mL/min)	2.2	2.5	3.6
Urine volume (mL/day)	900	600	350
D/P cr	0.77	0.75	0.57
PD prescriptions	1.36% glucose solution (4-h, 1 cycle) and icodextrin solution (8-h)	1.36% glucose solution (4-h, 2 cycles) and icodextrin solution (8-h)	1.36% glucose solution (4-h, 3 cycles) and icodextrin solution (8-h)
Medications	Nifedipine	Nifedipine	Nifedipine
	Furosemide	Olmesartan	Azilsartan
	Calcium carbonate	Calcium carbonate	Calcium carbonate
	Febuxostat	Atorvastatin	Lanthanum carbonate
	Warfarin		Alfacalcidol
	Hydroxyzine		Fexofenadine
	Epinastine		

wrKt/V: weekly renal Kt/V; wpKt/V: weekly peritoneal Kt/V; wtKt/V: weekly total Kt/V; rCcr: renal creatinine clearance; pCcr: peritoneal creatinine clearance; D/P cr: 4-h dialysate/plasma creatinine.

### Case 2

A 79-year-old woman with ESRD of unknown cause initiated PD in October 2012 using 1.36% glucose solution for a daytime exchange and icodextrin solution for a night exchange. She had complained of moderate skin itchiness for several years around the induction of PD, and diphenhydramine ointment and skin moisturizer had been prescribed. In addition, we had instructed the patient and her family on the appropriate skincare. However, she presented with systemic pruritic eruptions in May 2017. Although betamethasone ointment and oral epinastine were prescribed, her skin symptoms worsened by scratching. Systemic diffuse blisters and erosive skin lesions developed in the following 2 months (Figure 1(B)). The laboratory data, PD condition, and PD prescriptions are shown in Table 1. The autoantibody for BP was positive (anti-BP180-NC16a antibody 45.8 U/mL), whereas the autoantibodies for pemphigus were negative (anti-Dsg1 antibody <3.0 U/mL and anti-Dsg3 antibody <3.0 U/mL). Skin biopsy revealed subepidermal blister formation and multiple eosinophil infiltration accompanied by the deposition of BP-specific autoantibody. She was diagnosed with BP and administered oral PSL 1 mg/kg (40 mg) daily for 2 weeks. She obtained clinical remission of BP with the disappearance of blister formation and erosion. PSL was tapered (−5 mg/week) without recurrence. In November 2017, she was transferred to a nursing home due to age-related mobility disability and stopped visiting our hospital.

### Case 3

A 62-year-old man, who had been diagnosed with ESRD due to IgA nephropathy, started PD in October 2015 using 1.36% glucose solution for daytime exchanges and icodextrin solution for a night exchange. One year after the initiation of PD, he changed the disinfectant for the exit-site of his PD catheter from povidone–iodine solution to chlorhexidine gluconate solution because of his focal skin redness and itchiness. The skin symptoms partially improved. In April 2017, automated PD (APD) was introduced, because of the loss of residual renal function. Then, he presented with focal pruritic erosive lesions and small blisters at his left auricula and abdominal skin around the exit-site of his PD catheter (Figure 1(C)). The laboratory data, PD condition, and PD prescriptions at the onset of skin lesions are shown in Table 1. The autoantibody for BP was positive (anti-BP180-NC16a antibody 113.0 U/mL), whereas the autoantibodies for pemphigus were negative (anti-Dsg1 antibody <3.0 U/mL and anti-Dsg3 antibody <3.0 U/mL). The skin biopsy revealed subepidermal blister formation, eosinophilic change of epidermis, and eosinophilic and lymphocytic infiltration accompanied by the deposition of BP-specific autoantibody. He was diagnosed with BP and treated with hydrocortisone-bacterial culture suspension mixture ointment (Eksalb^®^) to improve the focal skin lesions for a month. He obtained clinical and immunological remission of BP by October 2018.

## Discussion

BP is a relatively rare autoimmune blistering skin disease. Immunologically, tissue-bound and circulating autoantibodies against two hemidesmosomal proteins of the basement membrane zone named BP230 (BPAG1) and BP180 (BPAG2), the latter most associated with disease activity, are observed in the patients [17,18]. BP typically occurs in elderly people (usually in late 70 s and 80 s), but is observed at all ages including children [1,2]. Although the mechanisms for the predominance of BP in elderly people have not been clarified, age-related changes in the immune system including a decrease in regulatory T cells have been considered to be involved [19,20]. In addition to the agedness, several clinical factors might be involved in the development of BP. Medications including furosemide, nifedipine, dipeptidyl peptidase-4 inhibitors, and other drugs have been reported to affect the disease development [3,21]. Comorbidities, such as hematological malignancies [22] and neurological disorders [23], have also been suggested to be involved in the etiology. Moreover, physical factors, such as insect bite, irradiation, mechanical trauma, surgical wound, thermal burn, and UV exposure can be a trigger of the onset of BP [24].

In the three patients of this case series study, several aforementioned factors related to the onset of BP were found. First, the skin injury was observed in all the three cases. Indeed, pruritus and consequent scratching skin damage were observed in Cases 1 and 2, and disinfectant-induced chronic skin inflammation around the PD catheter exit-site was seen in Case 3. Pruritus is one of the most common clinical symptoms in patients with chronic kidney disease (CKD) [25]. The prevalence of pruritus has been reported to be 19% in CKD patients, independent from the stage of CKD [26], and up to 84% in patients receiving maintenance dialysis therapy [27]. The time interval between the exposure to the trigger and the development of BP is usually less than a year [24]. In our cases, the skin injury had been observed shortly before the onset of BP at least in Cases 1 and 3 (several months), whereas the duration between the skin injury and the disease onset was several years in Case 2. Although the mechanisms whereby the skin injury induces BP have not been clarified yet, there are several hypotheses. For example, the preclinical existence of non-pathogenic low titers of anti-hemidesmosomal autoantibodies accompanied by tissue damage would contribute to the autoantibody-mediated inflammatory reaction followed by the onset of BP [28]. Various manifestations of skin injury, in combination with several potential clinical factors including certain medications and ESRD-related immunological disturbance described later, would participate in the development of BP.

Multiple drugs have been suggested to affect the development of BP [3,21]. In our cases, furosemide was prescribed in Case 1, whereas nifedipine was prescribed in all the three cases. These medications might be a potential trigger of the disease development, although they had been prescribed for more than years in all the cases. Moreover, icodextrin has been reported to induce allergic skin disorders, such as rash or pruritus [29], thus, it would also affect the onset of BP. The exposure to icodextrin was a common factor among the three cases. Indeed, we suspected icodextrin exposure to be a cause of the skin lesion in Case 1, but the skin lesion was not improved by the discontinuation of the prescription of icodextrin. In Cases 2 and 3, we suspected allergic reactions to icodextrin exposure accounting for the skin lesions at early time points. We performed drug-induced lymphocyte stimulation tests to eliminate possibilities of an allergic reaction to icodextrin in these two cases, although the results were negative.

We reviewed previously reported 14 dialysis patients (12 with HD and 2 with PD) developing BP, and compared with our three cases with PD (Table 2). Among the five PD cases, the skin injury was suspected to affect the onset of BP in four cases (exit-site-related skin damage in No. 14 and 17 and chronic scratching skin damage in Nos. 15 and 16) [16]. In the rest one case, phototoxicity of furosemide, followed by inflammatory skin damage, was a possible trigger of the onset (No. 8) [15]. Various skin damage would account for the onset of BP in PD patients. In HD cases, the use of vascular access, including arteriovenous fistula, vascular graft, and central venous line, was suspected to evoke skin injury, resulting in the onset of BP (Nos. 2, 4, 5, 6, and 12) [5,7,8,13]. In Nos. 9 and 13, renal allograft rejection might induce tissue destruction and subsequent activation of autoimmunity which affected the onset of BP, in combination with secession of immunosuppressants [10,14]. Renal allograft rejection, rather than the withdrawal of immunosuppressants, was suspected to be related to the onset of BP, based on a renal transplant case obtaining remission of BP after the removal of rejected renal allograft [30]. Interestingly, allergic reaction to dialyzer membrane would also be responsible for the onset (No. 10) [11]. In all cases, ESRD-associated immunological abnormalities would be a fundamental factor of the etiology.

**Table 2.** Clinical characteristics of dialysis patients who developed BP.

No.	Author	Age (years)	Sex	Race	Etiology of ESRD	HD/PD	Dialysis History	Trigger of BP
1	Sato et al. [4]	43	Male	Asian	Unknown	HD	17 years	Sun exposure
2	Freeman and Rubin [5]	72	Female	African American	DM	HD	At initiation	Polytetrafluoroethylene (PTFE) vascular graft placement
3	Kamada et al. [6]	73	Male	Asian	Hydronephrosis	HD	8 years	Unknown
4	Yasudian et al. [7]	73	Male	Caucasian	Renal sclerosis	HD	4 years	Skin injury induced by vascular access and infraclavicular central venous line
5	Pardo et al. [8]	76	Male	Caucasian	DM	HD	7 years	Skin injury induced by vascular access
6	Pardo et al. [8]	52	Female	Caucasian	Pyelonephritis	HD	9 years	Skin injury induced by vascular access
7	Seneschal et al. [9]	76	Male	Caucasian	Renal sclerosis	HD	2 years	Unknown
8	Takeichi et al. [15]	58	Male	Asian	Unknown	PD	4 years	Combination of furosemide administration and sun exposure
9	Peruzzo et al. [10]	15	Female	Caucasian	CGN	HD^a^	4 months	Chronic renal allograft rejection
10	Sodemoto et al. [11]	75	Female	Caucasian	CGN	HD	4 years	Allergic reaction to dialyzer membrane (cellulose triacetate)
11	Liu et al. [12]	48	Male	Asian	CGN	HD	5 years	Unknown
12	Osipowicz et al. [13]	33	Male	Caucasian	Renal sclerosis	HD^b^	1 year	Skin injury induced by vascular access
13	Abdul Salim et al. [14]	45	Female	African American	DM	HD^c^	10 months	Chronic renal allograft rejection and discontinuation of immunosuppressants
14	Giunzioni [16]	76	Male	Caucasian	DM	PD	1 year	Skin injury (PD catheter placement and daily plaster removal)
15	Current Case 1	72	Male	Asian	Unknown	PD	1 month	Skin injury (scratching skin damage)
16	Current Case 2	79	Female	Asian	Unknown	PD	5 years	Skin injury (scratching skin damage)
17	Current Case 3	62	Male	Asian	IgA nephropathy	PD	2 years	Skin injury (daily disinfectant application)

ESRD: end-stage renal disease; DM: diabetes mellitus; CGN: chronic glomerulonephritis.

^a^HD for 3 years, renal transplantation 10 years before, ESRD due to chronic renal allograft rejection, and HD for 4 months.

^b^HD for 1 year, renal transplantation 7 years before, ESRD due to chronic renal allograft rejection, and HD for 1 year.

^c^Renal transplantation 16 years before, ESRD due to chronic renal allograft rejection, and HD for 10 months.

The incidence of BP in ESRD patients has not been reported yet. The annual incidence of BP has been reported to be 2.5–42.8/million in Europe [30]. Two independent epidemiological investigations in Germany and France have revealed that the incidence of BP is increasing mainly because of the development of diagnostic procedures and the aging in the general population [30]. Of interest, the incidence of BP in our PD patients was 3/478.4 person-years, as determined by the 10-year observation (from April 2010 to March 2020) of 130 PD patients who regularly visited our hospital. Although this is a retrospective single-center survey, the incidence of BP in PD patients seems to be much higher than those reported in the general population. The influence of ESRD or dialysis therapy on the development of BP remains to be elucidated. Although the reduction of regulatory T cells has been reported to affect the predominance of BP in elderly population [19,20], circulating regulatory T cells were not decreased in ESRD patients, compared with healthy subjects [31]. In addition, circulating regulatory T cell level was independent of glomerular filtration rate and serum levels of urea nitrogen and creatinine [31]. Meanwhile, peritoneal T cell balance would be affected by PD in experimental animal models. PD and PD-induced peritoneal stimulation have been reported to induce T helper 17 cells and reciprocally reduce regulatory T cells [32]. Clarification of the detailed mechanisms whereby BP occurs at a high incidence rate in ESRD or dialysis patients is required in future.

## Conclusion

We presented three PD patients developing BP and conducted a literature survey on dialysis patients complicated with BP. Our survey has revealed that the skin and tissue injuries, such as vascular access-related skin damage, chronic scratching skin damage, and renal allograft rejection, may be formerly unappreciated triggers for the onset of BP in dialysis patients. The incidence of BP in PD patients might be much higher than that in the general population.
